# Supplementary material for: The LOTUS initiative for open knowledge management in natural products research
Source: eLife. 2022 May 26;11:e70780. doi: 10.7554/eLife.70780 (PMC9135406; doi:10.7554/eLife.70780)
Supplement: Supplementary file 1. [file elife-70780-supp1.docx]

### Supplementary file 1. Wikidata Queries

#### Query 1 - *Arabidopsis thaliana*

This query answers to the following question:

What are the compounds found in Mouse-ear cress Arabidopsis thaliana (Q158695) or children taxa?

Link: <https://w.wiki/4Vcv>

#title: What are the compounds found in Mouse-ear cress Arabidopsis thaliana (Q158695) or children taxa?
SELECT DISTINCT ?structure ?structureLabel ?structure_inchi WHERE {
 VALUES ?taxon {
 wd:Q158695 # You can remove the Qxxxxxx and hit Ctrl+space, type the first letters and it should autocomplete
 }
 ?children (wdt:P171*) ?taxon. # Include children taxa
 ?structure wdt:P234 ?structure_inchi; # Get the InChI
 (p:P703/ps:P703) ?children. # Found in given taxon/taxa
 SERVICE wikibase:label { bd:serviceParam wikibase:language "en". }
}

#### Query 2 - *β*-sitosterol

This query answers to the following question:

Which organisms are known to contain β-sitosterol?

Link: <https://w.wiki/4VFn>

#title: Which organisms are known to contain Beta-Sitosterol (Q121802)?
SELECT DISTINCT ?taxon ?taxon_name WHERE {
 VALUES ?compound {
 wd:Q121802 # You can remove the Qxxxxxx and hit Ctrl+space, type the first letters and it should autocomplete
 }
 ?compound (p:P703/ps:P703) ?taxon. # Found in taxon
 ?taxon wdt:P225 ?taxon_name. # Get scientific name of the taxon
 SERVICE wikibase:label { bd:serviceParam wikibase:language "en". }
}

#### Query 3 - *β*-sitosterol stereoisomers

This query answers to the following question:

Which organisms are known to contain which stereoisomers of β-sitosterol?

Link: <https://w.wiki/4VFq>

#title: Which organisms are known to contain stereoisomers of Beta-Sitosterol (Q121802)?
SELECT ?taxon_name ?compound ?InChIKey
WITH {
 SELECT ?compound ?InChIKey WHERE {
 VALUES ?target {
 wd:Q121802
 }
 ?target wdt:P235 ?queryKey.
 ?compound wdt:P235 ?InChIKey.
 FILTER (REGEX(STR(?InChIKey), CONCAT("^", SUBSTR($queryKey,1,14), "-")))
 FILTER (?InChIKey != ?queryKey)
 }
} AS %compounds
WHERE {
 INCLUDE %compounds
 ?compound (wdt:P703/wdt:P225) ?taxon_name.
 SERVICE wikibase:label { bd:serviceParam wikibase:language "en". }
}

#### Query 4 - Pigments

This query answers to the following question:

Which pigments are found in which taxa, according to which reference?

Link: <https://w.wiki/4VFx>

#title: Which pigments are found in which taxa, according to which reference?
# special thanks goes to User:Lmichan for updating this information!
SELECT DISTINCT ?compound ?compoundLabel ?taxon ?taxonname ?DOI
WITH {
 SELECT ?compound WHERE {
 ?compound (wdt:P31*/wdt:P279*) wd:Q161179. # get pigments
 }
} AS %compounds
WITH {
 SELECT ?compound ?P703statement WHERE {
 INCLUDE %compounds
 ?compound p:P703 ?P703statement. # check for "found in taxon" statements
 }
} AS %P703statement
WITH {
 SELECT ?compound ?taxon ?DOI WHERE {
 INCLUDE %P703statement
 ?P703statement ps:P703 ?taxon ; # get the respective taxa
 prov:wasDerivedFrom / pr:P248 [ # get the reference supporting that statement
 wdt:P356 ?DOI # get the DOI for the reference
 ] .
 }
} AS %taxa
WHERE {
 {
 INCLUDE %taxa

 ?taxon wdt:P225 ?taxonname . # get the taxon name
 }
 ?compound rdfs:label ?compoundLabel . # get compound labels
 FILTER (LANG(?compoundLabel) = "en") . # filter for English
}
ORDER BY ASC(?compoundLabel)
LIMIT 10000

#### Query 5 - Sister taxon compounds

This query answers to the following question:

What are examples of organisms where compounds were found in an organism sharing the same parent taxon, but not in the organism itself?

Link: <https://w.wiki/4Wt3>

#title: What are examples of organisms where compounds were found in an organism sharing the same parent taxon, but not the organism itself?
SELECT DISTINCT ?compound ?compoundLabel ?taxonname_with_compound ?taxonname_without_compound ?parent_taxon WITH{
 SELECT DISTINCT ?compound ?taxon_with_compound ?parent_taxon
 WHERE {
 ?compound wdt:P235 ?inchikey.
 SERVICE bd:sample { ?compound wdt:P703 ?taxon_with_compound. bd:serviceParam bd:sample.limit 1000 }
 ?taxon_with_compound wdt:P171 ?parent_taxon.
 }
} AS %taxon_with_compound
WITH
{
 SELECT DISTINCT ?taxon_without_compound ?parent_taxon ?compound
 WHERE {
 INCLUDE %taxon_with_compound
 ?taxon_without_compound wdt:P171 ?parent_taxon.
 FILTER (?taxon_with_compound != ?taxon_without_compound)
 }
} AS %taxon2
WHERE {
 INCLUDE %taxon_with_compound
 INCLUDE %taxon2
 FILTER NOT EXISTS {?compound wdt:P703 ?taxon_without_compound.}
 ?taxon_with_compound wdt:P225 ?taxonname_with_compound.
 ?taxon_without_compound wdt:P225 ?taxonname_without_compound.
 ?compound rdfs:label ?compoundLabel.
 FILTER(LANG(?compoundLabel) = "en").
}

#### Query 6 - *Zephyranthes* sister taxon compounds

This query answers to the following question:

Which Zephyranthes spp. lack compounds known from ≥ 2 other Zephyranthes?

Link: <https://w.wiki/4VG3>

#title: Which Zephyranthes (Q191364) spp. lack compounds known from at least two species in the genus?
PREFIX target: <http://www.wikidata.org/entity/Q191364> # Zephyranthes
SELECT DISTINCT ?compound ?compoundLabel ?taxon_with_compound ?another_taxon_with_compound ?taxon_without_compound WITH {
 SELECT DISTINCT ?compound ?taxon_YES_1 ?taxon_YES_2
 WHERE {
 ?compound wdt:P703 ?taxon_YES_1 .
 ?compound wdt:P703 ?taxon_YES_2 .
 ?taxon_YES_1 wdt:P171 target: .
 ?taxon_YES_2 wdt:P171 target: .
 FILTER (?taxon_YES_2 != ?taxon_YES_1)
 }
} AS %taxa_with_compound
WITH
{
 SELECT DISTINCT ?taxon_NO ?compound
 WHERE {
 INCLUDE %taxa_with_compound
 ?taxon_NO wdt:P171 target: .
 FILTER (?taxon_YES_1 != ?taxon_NO)
 }
} AS %taxon_without_compond
WHERE {
 INCLUDE %taxa_with_compound
 INCLUDE %taxon_without_compond
 FILTER NOT EXISTS { ?compound wdt:P703 ?taxon_NO .}
 VALUES ?classes {
 wd:Q11173
 wd:Q59199015
 }
 ?taxon_YES_1 wdt:P225 ?taxon_with_compound .
 ?taxon_YES_2 wdt:P225 ?another_taxon_with_compound .
 ?taxon_NO wdt:P225 ?taxon_without_compound .
 ?compound (wdt:P31*/wdt:P279*) ?classes .
 ?compound rdfs:label ?compoundLabel.
 FILTER(LANG(?compoundLabel) = "en").
}

#### Query 7 - Antibiotic-like compounds

This query answers to the following question:

How many compounds are structurally similar to compounds labeled as antibiotics? Results are grouped by the parent taxon of the organism they were found in.

Link: <https://w.wiki/4VG4>

#title: How many compounds are structurally similar to compounds labeled as antibiotics? Results are grouped by the parent taxon of the organism they were found in.
PREFIX sachem: <http://bioinfo.uochb.cas.cz/rdf/v1.0/sachem#> # prefixes needed for structural similarity search
PREFIX idsm: <https://idsm.elixir-czech.cz/sparql/endpoint/>
SELECT ?parent_taxon ?parent_taxon_name (COUNT(DISTINCT ?compound) AS ?count) WHERE {
 SERVICE idsm:wikidata {
 VALUES ?CUTOFF {
 "0.9"^^xsd:double
 }
 SERVICE <https://query.wikidata.org/bigdata/namespace/wdq/sparql> {
 VALUES ?MESH {
 "D000900"
 }
 ?antibiotic ((wdt:P279*)/wdt:P2868/wdt:P486) ?MESH;
 wdt:P233 ?smiles.
 }
 ?compound sachem:similarCompoundSearch _:b40.
 _:b40 sachem:query ?smiles;
 sachem:cutoff ?CUTOFF.
 }
 hint:Prior hint:runFirst "true"^^xsd:boolean.
 ?compound wdt:P703 ?taxon.
 ?taxon wdt:P171 ?parent_taxon.
 OPTIONAL { ?parent_taxon wdt:P225 ?parent_taxon_name. }
 SERVICE wikibase:label { bd:serviceParam wikibase:language "en". }
}
GROUP BY ?parent_taxon ?parent_taxon_name
ORDER BY DESC (?count)

#### Query 8 - Indolic scaffold

This query answers to the following question:

Which organisms contain indolic scaffolds? Count occurrences, group and order the results by the parent taxon.

Link: <https://w.wiki/4VG9>

#title: Which organisms contain indolic scaffolds? Count occurences, group and order the results by the parent taxon.
PREFIX sachem: <http://bioinfo.uochb.cas.cz/rdf/v1.0/sachem#> # prefixes needed for structural similarity search
PREFIX wd: <http://www.wikidata.org/entity/>
PREFIX p: <http://www.wikidata.org/prop/>
PREFIX idsm: <https://idsm.elixir-czech.cz/sparql/endpoint/>
SELECT ?parent_taxon ?parent_taxon_name (COUNT(DISTINCT ?compound) AS ?count) WHERE {
 SERVICE idsm:wikidata {
 VALUES ?SUBSTRUCTURE {
 "NCCC1=CNC2=C1C=CC=C2" # indolic scaffold
 }
 ?compound sachem:substructureSearch _:b16.
 _:b16 sachem:query ?SUBSTRUCTURE.
 }
 hint:Prior hint:runFirst "true"^^xsd:boolean.
 ?compound p:P703 ?statement;
 wdt:P235 ?inchikey.
 ?statement ps:P703 ?taxon.
 ?taxon wdt:P171 ?parent_taxon.
 ?parent_taxon wdt:P225 ?parent_taxon_name.
 SERVICE wikibase:label { bd:serviceParam wikibase:language "en". }
}
GROUP BY ?parent_taxon ?parent_taxon_name
ORDER BY DESC (?count)

#### Query 9 - Bioactive compounds in Actinobacteria

This query answers to the following question:

Which compounds with known bioactivities were isolated from Actinobacteria, between 2014 and 2019, with related organisms and references?

Link: <https://w.wiki/4VGC>

#title: Which compounds with known bioactivities were isolated from Actinobacteria (Q26262282), between 2014 and 2019, with related organisms and references?
SELECT ?organism ?organism_name ?compound ?compound_smiles (GROUP_CONCAT(DISTINCT ?meshLabel; SEPARATOR = "|") AS ?bioactivities) ?isolation_reference ?reference_title WHERE {
 ?organism (wdt:P171*) wd:Q26262282;
 wdt:P225 ?organism_name.
 ?compound wdt:P235 ?compound_id;
 wdt:P233 ?compound_smiles;
 p:P703 ?statement;
 (wdt:P2868/wdt:P486) ?meshId.
 ?mesh wdt:P486 ?meshId;
 rdfs:label ?meshLabel.
 FILTER(LANGMATCHES(LANG(?meshLabel), "EN"))
 ?statement ps:P703 ?organism;
 prov:wasDerivedFrom ?ref.
 ?ref pr:P248 ?isolation_reference.
 ?isolation_reference wdt:P1476 ?reference_title;
 wdt:P356 ?reference_doi;
 wdt:P577 ?reference_date.
 FILTER(((YEAR(?reference_date)) >= 2014 ) && ((YEAR(?reference_date)) <= 2019 ))
}
GROUP BY ?organism ?organism_name ?compound ?compound_smiles ?isolation_reference ?reference_title
LIMIT 100000

#### Query 10 - *Aspergillus* spp. terpenoids

This query answers to the following question:

Which compounds labeled as terpenoids were found in Aspergillus spp., between 2010 and 2020, with related references?

Link: <https://w.wiki/4VGD>

#title: Which compounds labelled as terpenoid (Q426694) were found in Aspergillus (Q335130) spp., between 2010 and 2020, with related references?
SELECT ?compound ?compound_inchi (GROUP_CONCAT(DISTINCT ?isolation_reference; SEPARATOR = "|") AS ?isolation_references) (GROUP_CONCAT(DISTINCT ?reference_title; SEPARATOR = "|") AS ?references_titles) WHERE {
 VALUES ?taxon {
 wd:Q335130
 }
 VALUES ?chemical_class {
 wd:Q426694
 }
 ?organism (wdt:P171*) ?taxon.
 ?compound wdt:P235 ?compound_id;
 wdt:P234 ?compound_inchi;
 ((wdt:P31|wdt:P279)/(wdt:P279*)) ?compound_class;
 p:P703 ?statement.
 ?statement ps:P703 ?organism;
 (prov:wasDerivedFrom/pr:P248) ?isolation_reference.
 ?isolation_reference wdt:P1476 ?reference_title;
 wdt:P356 ?reference_doi;
 wdt:P577 ?reference_date.
 FILTER(((YEAR(?reference_date)) >= 2010 ) && ((YEAR(?reference_date)) <= 2020 ))
 FILTER(?compound_class = ?chemical_class)
}
GROUP BY ?compound ?compound_inchi

#### Query 11 - Triples

This query answers to the following question:

Which are the available referenced structure-organism pairs on Wikidata? (example limited to 1000 results)

Link: <https://w.wiki/4VFh>

#title: Which are the available referenced structure-organism pairs on Wikidata? (example limited to 1000 results)
SELECT DISTINCT ?structure ?structure_inchikey ?taxon ?taxon_name ?reference ?reference_doi WHERE {
 ?structure wdt:P235 ?structure_inchikey; # get the inchikey
 p:P703[ # statement found in taxon
 ps:P703 ?taxon; # get the taxon
 (prov:wasDerivedFrom/pr:P248) ?reference ]. # get the reference
 ?taxon wdt:P225 ?taxon_name. # get the taxon scientific name
 ?reference wdt:P356 ?reference_doi. # get the reference DOI
}
LIMIT 1000
