# Supplementary material for: The LOTUS initiative for open knowledge management in natural products research
Source: eLife. 2022 May 26;11:e70780. doi: 10.7554/eLife.70780 (PMC9135406; doi:10.7554/eLife.70780)
Supplement: Supplementary file 2. [file elife-70780-supp2.docx]

### Supplementary file 2. Wikidata Entry Creation Tutorial

**Tutorial for manual creation** also check <https://lotus.nprod.net/post/tuto-wd-entry-creation> for the latest version of this tutorial.

#### Manual addition of a referenced structure-organism pair to Wikidata

##### Select a referenced structure-organism pair

Throughout this demonstration, we are going to use the following example: > [trigocherrin A](https://pubchem.ncbi.nlm.nih.gov/compound/101556657) is found in [*Trigonostemon cherrieri*](http://gni.globalnames.org/name_strings?search_term=trigonostemon+cherrieri&commit=Search), as stated in [Trigocherrin A, the first natural chlorinated daphnane diterpene orthoester from Trigonostemon cherrieri](https://doi.org/10.1021/OL2030907).

##### Fetch the information for the referenced structure-organism pair

###### Structure

Search PubChem for your compound, here [trigocherrin A](https://pubchem.ncbi.nlm.nih.gov/#query=%22trigocherrin%20A%22). This leads to <https://pubchem.ncbi.nlm.nih.gov/compound/101556657>.


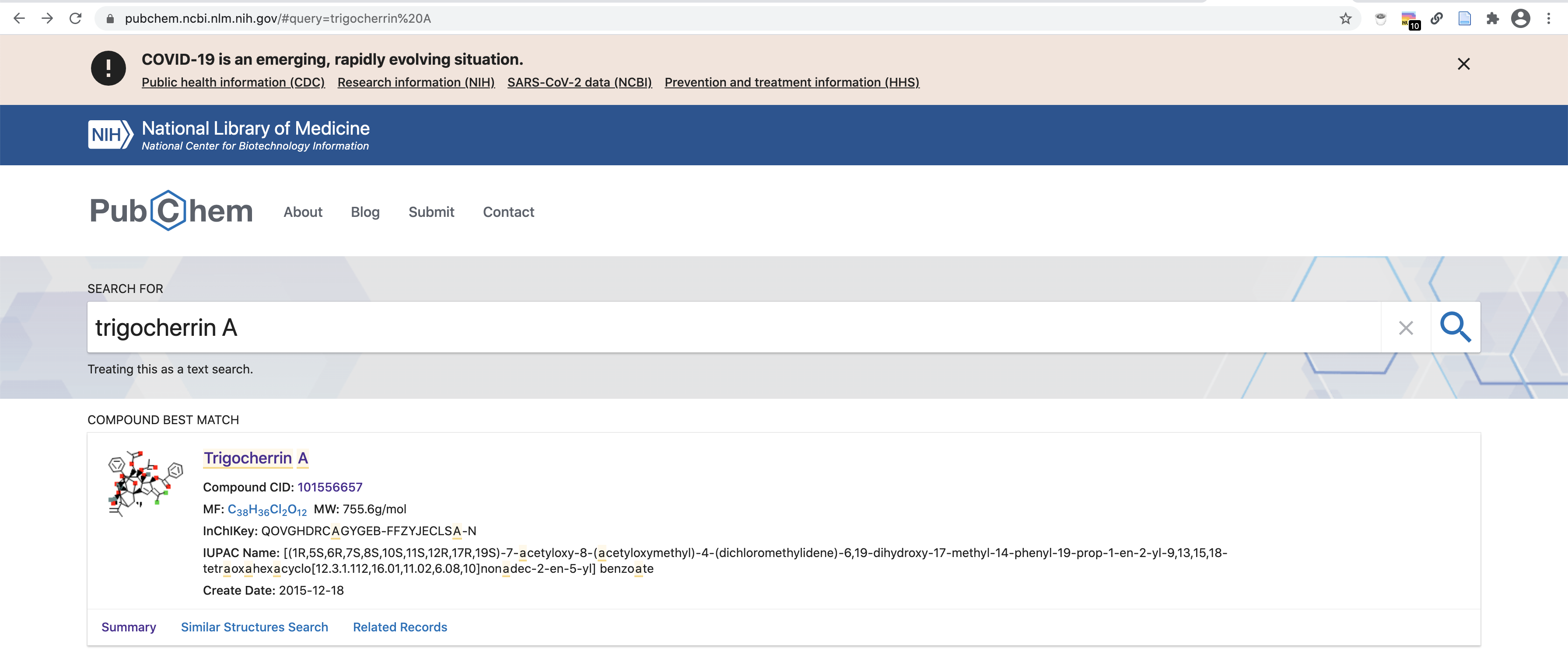


From there, you can fetch the compound’s name, InChIKey and InChI as well as its Canonical and Isomeric SMILES. Here we keep, respectively:

- trigocherrin A
- QOVGHDRCAGYGEB-FFZYJECLSA-N
- InChI=1S/C38H36Cl2O12/c1-18(2)35(44)27-19(3)37-25-16-24(31(39)40)28(48-32(43)22-12-8-6-9-13-22)36(25,45)33(47-21(5)42)34(17-46-20(4)41)29(49-34)26(37)30(35)51-38(50-27,52-37)23-14-10-7-11-15-23/h6-16,19,26-30,33,44-45H,1,17H2,2-5H3/t19-,26+,27?,28+,29+,30-,33-,34+,35+,36-,37+,38?/m1/s1
- CC1C2C(C3C4C1(C5=CC(=C(Cl)Cl)C(C5(C(C6(C4O6)COC(=O)C)OC(=O)C)O)OC(=O)C7=CC=CC=C7)OC(O2)(O3)C8=CC=CC=C8)(C(=C)C)O
- C[C@@H]1C2[C@]([C@H]3[C@H]4[C@]1(C5=CC(=C(Cl)Cl)[C@@H]([C@]5([C@@H]([C@@]6([C@H]4O6)COC(=O)C)OC(=O)C)O)OC(=O)C7=CC=CC=C7)OC(O3)(O2)C8=CC=CC=C8)(C(=C)C)O

###### Organism

You can check if your organism name is correctly spelled using the Global Names resolver service: <http://gni.globalnames.org/name_strings?search_term=trigonostemon+cherrieri&commit=Search>.


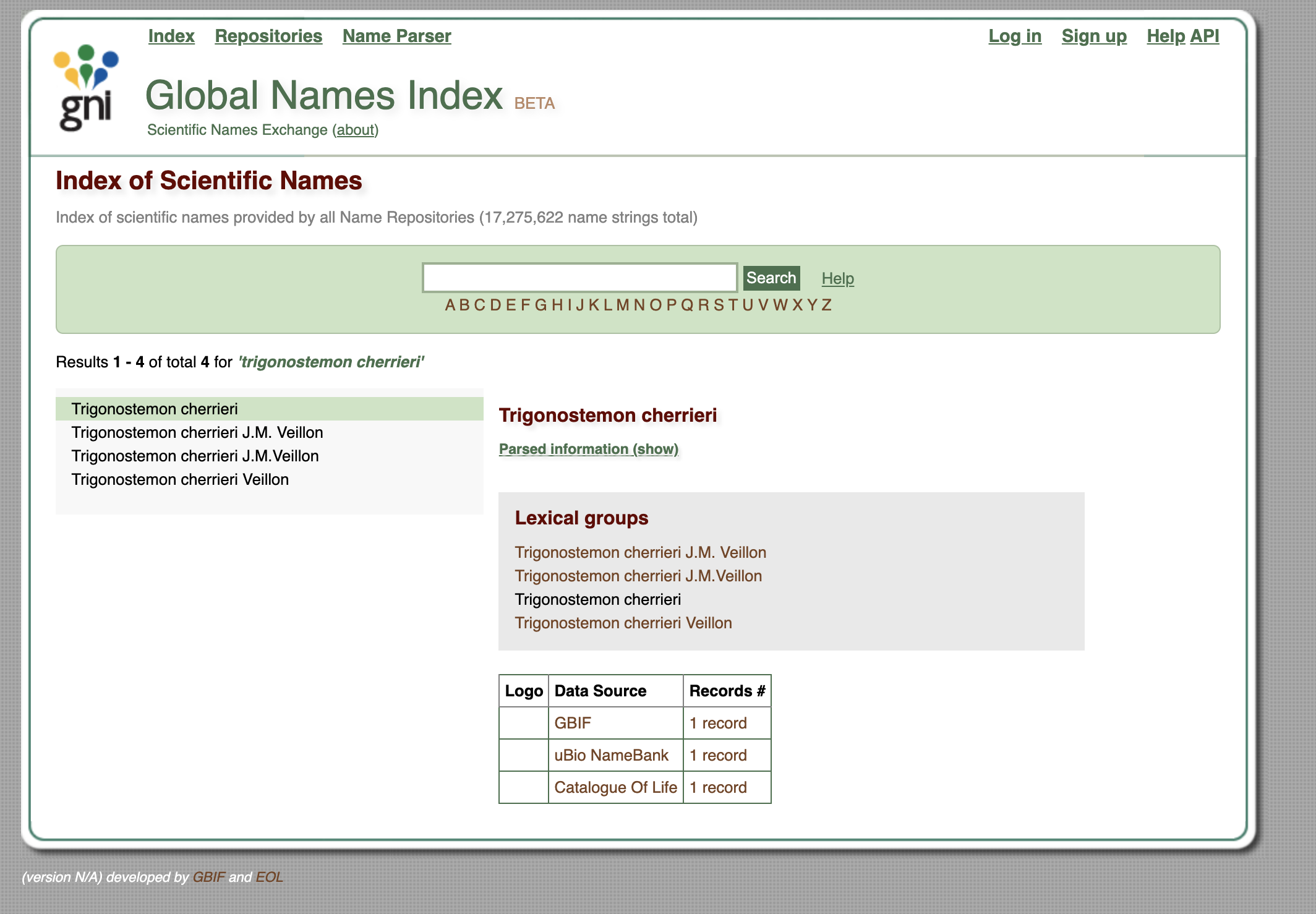


Alternatively, you can use [gnfinder](https://github.com/gnames/gnfinder) in your command line interface to check for the spelling of your organism string.

echo "Trigonostemion cherrieri" | gnfinder find -c -l eng
{
 "metadata": {
 "date": "2021-02-27T18:44:41.640982+01:00",
 "gnfinderVersion": "v0.11.1",
 "withBayes": true,
 "tokensAround": 0,
 "language": "eng",
 "detectLanguage": false,
 "totalWords": 2,
 "totalCandidates": 1,
 "totalNames": 1
 },
 "names": [
 {
 "cardinality": 2,
 "verbatim": "Trigonostemion cherrieri",
 "name": "Trigonostemion cherrieri",
 "odds": 77581.46698350731,
 "start": 0,
 "end": 24,
 "annotationNomenType": "NO_ANNOT",
 "annotation": "",
 "verification": {
 "bestResult": {
 "dataSourceId": 1,
 "dataSourceTitle": "Catalogue of Life",
 "taxonId": "1575885",
 "matchedName": "Trigonostemon cherrieri Veillon",
 "matchedCardinality": 2,
 "matchedCanonicalSimple": "Trigonostemon cherrieri",
 "matchedCanonicalFull": "Trigonostemon cherrieri",
 "classificationPath": "Plantae|Tracheophyta|Magnoliopsida|Malpighiales|Euphorbiaceae|Trigonostemon|Trigonostemon cherrieri",
 "classificationRank": "kingdom|phylum|class|order|family|genus|species",
 "classificationIds": "3939764|3942634|3942724|3942777|3942795|4210752|1575885",
 "editDistance": 1,
 "stemEditDistance": 1,
 "matchType": "FuzzyCanonicalMatch"
 },
 "dataSourcesNum": 13,
 "dataSourceQuality": "HasCuratedSources",
 "retries": 1
 }
 }
 ]
}

For misspellings like *Trigonstemion cherrieri*, gnfinder can help resolve them, in this case to *Trigonostemon cherrieri*.

###### Reference

Make sure that you have the correct [Digital Object Identifier (DOI)](https://www.doi.org/doi_handbook/Glossary.html#doi) for it. For “[Trigocherrin A, the first natural chlorinated daphnane diterpene orthoester from Trigonostemon cherrieri](https://doi.org/10.1021/OL2030907)”, this is **10.1021/ol2030907**. Note that DOIs are uppercase-normalized in Wikidata.

##### Check for the presence of your compound in Wikidata

Using the compound’s InChIKey (i.e. QOVGHDRCAGYGEB-FFZYJECLSA-N for trigocherrin A), run a SPARQL query to check if your compound is present in Wikidata or not:

SELECT ?item ?itemLabel WHERE {
 VALUES ?classes {
 wd:Q11173 # chemical compound
 wd:Q59199015 # group of stereoisomers
 wd:Q79529 # chemical substance
 wd:Q17339814 # group of chemical substances
 wd:Q47154513 # structural class of chemical compounds
 }
 ?item wdt:P31 ?classes. # instance of
 ?item wdt:P235 'QOVGHDRCAGYGEB-FFZYJECLSA-N'
 SERVICE wikibase:label { bd:serviceParam wikibase:language "[AUTO_LANGUAGE],en". }
}

[Try this query](https://w.wiki/32zU). You can adapt it by replacing the InChIKey with the one for your compound.

Alternatively you can use the following Scholia link (replace by your compounds InChIKey) <https://scholia.toolforge.org/inchikey/QOVGHDRCAGYGEB-FFZYJECLSA-N>

If your compound is already present on Wikidata, you can directly skip to the [Add the biological source information](#add_bio) section below.

##### Add your data manually to Wikidata

First, if you do not have a Wikidata account already, it is advisable that you create one via <https://www.wikidata.org/wiki/Special:CreateAccount>. While an account is not strictly required for manual edits, having one will be useful if you want to contribute more than once, and it helps in getting your contributions recognized. Note that Wikidata accounts are integrated with accounts across the Wikimedia ecosystem, so if you already have an account on, say, any Wikipedia or on Wikispecies, then you can use the same credentials on Wikidata.

If you are unfamiliar with how Wikidata works, you can start by reading the Wikidata introduction page <https://www.wikidata.org/wiki/Wikidata:Introduction> and have a look at the Wikidata Tours page <https://www.wikidata.org/wiki/Wikidata:Tours>.

Now that you are all set up, you can go to Wikidata’s page for creating new items, <https://www.wikidata.org/wiki/Special:NewItem>:


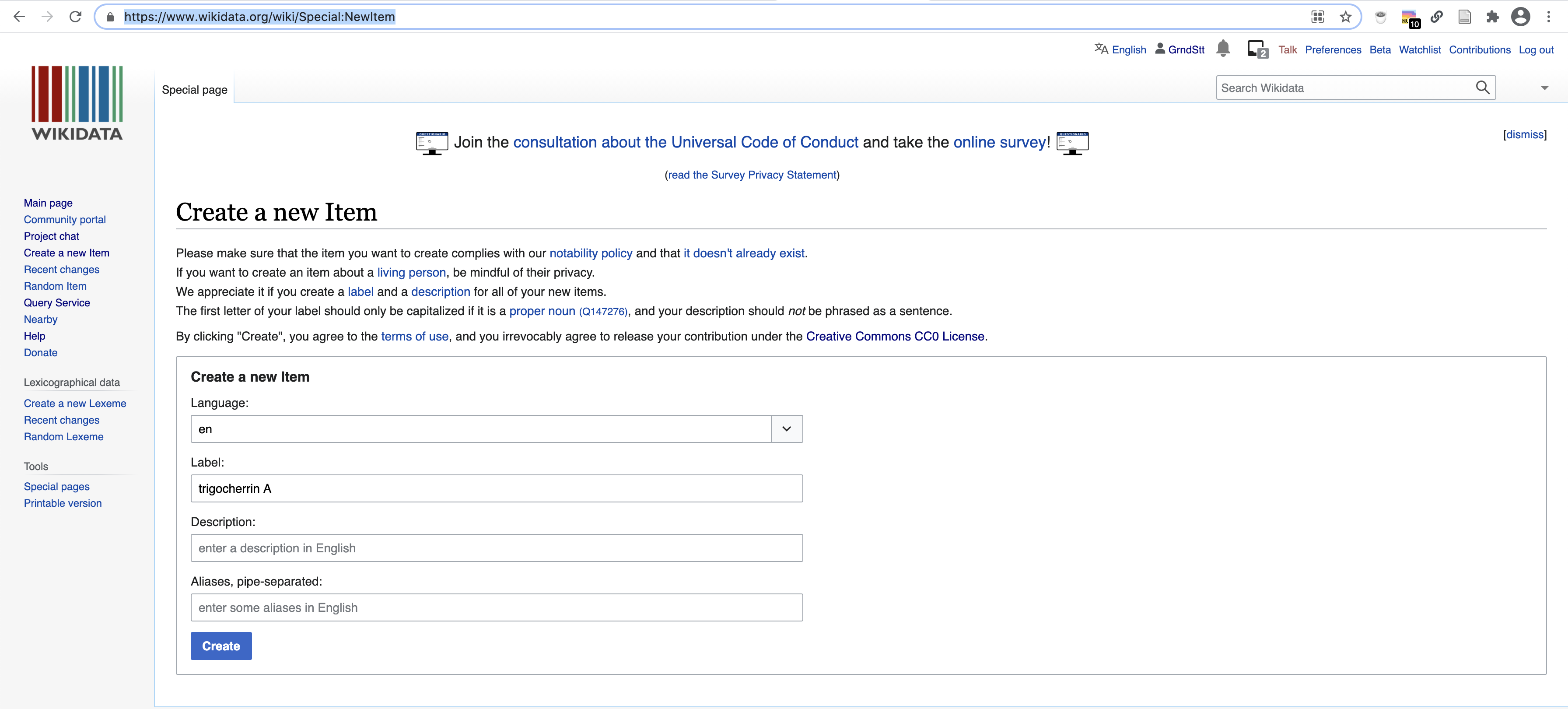


An empty page with a new Wikidata identifier is created


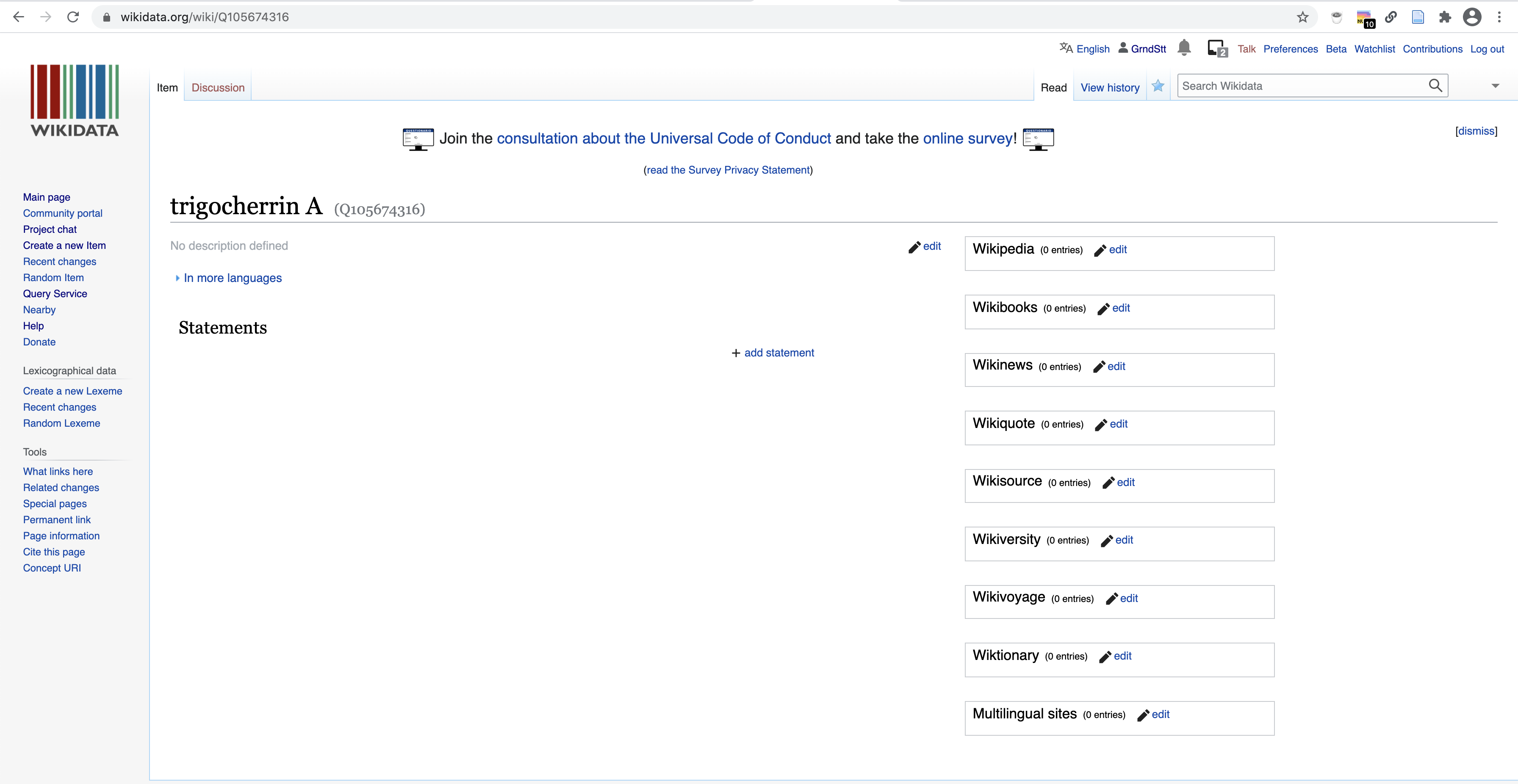


###### Add the chemical compound information

Create a new statement for is an instance of


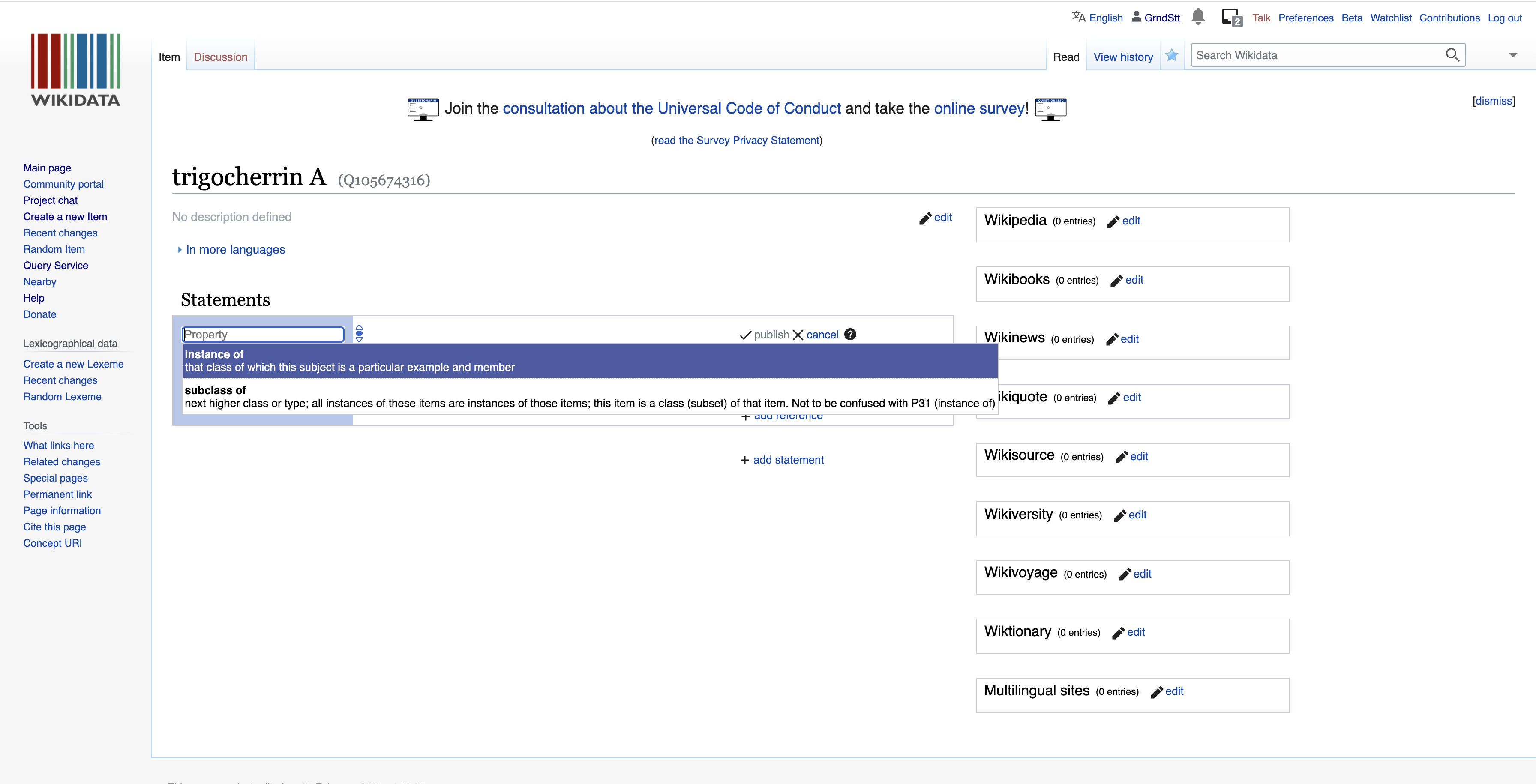


and select chemical compound (i.e. [Q11173](https://www.wikidata.org/wiki/Q11173)):


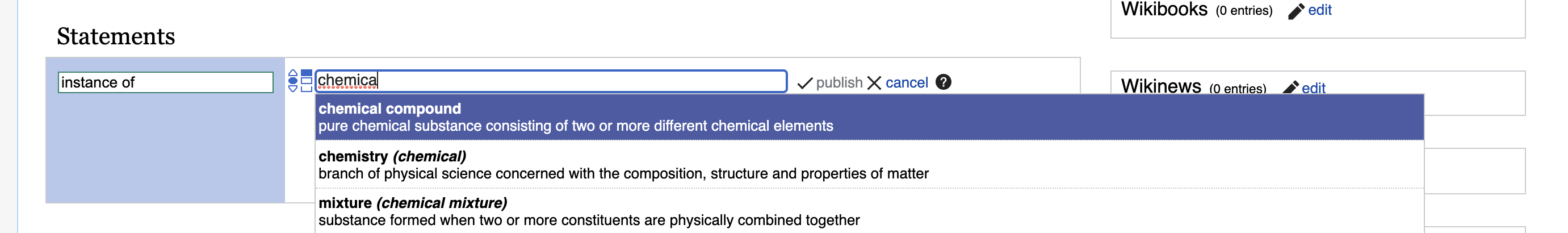


Click publish to save your changes and make them public.

Since you created a new item about an instance of a chemical compound, the user interface will automatically propose to you a set of additional statements commonly found on items about chemical compounds.


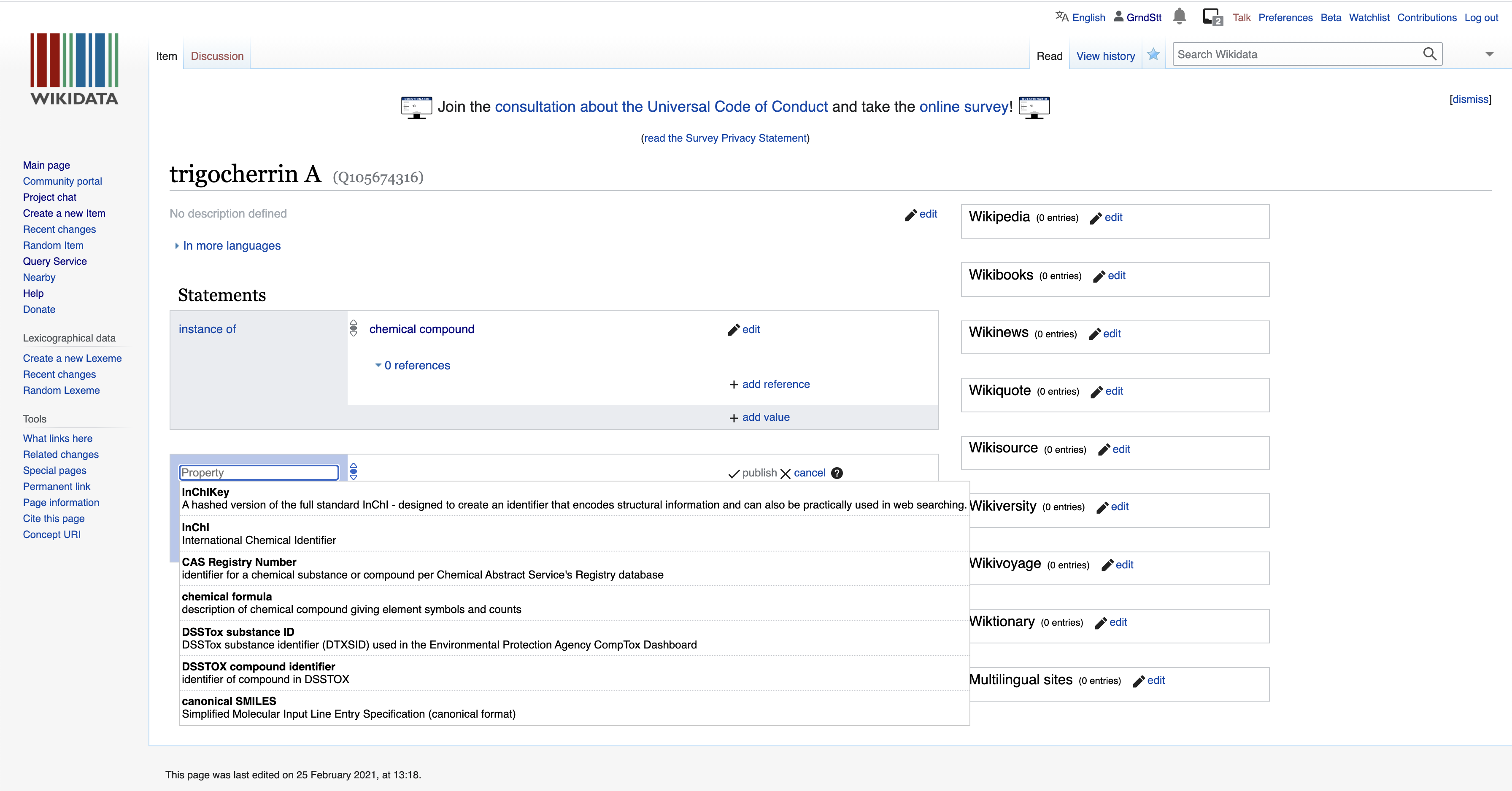


You can then go on and fill these in.

Here, we start with the InChIKey. Note the little flag which will automatically tell you if you have some problems with the recently created statements.


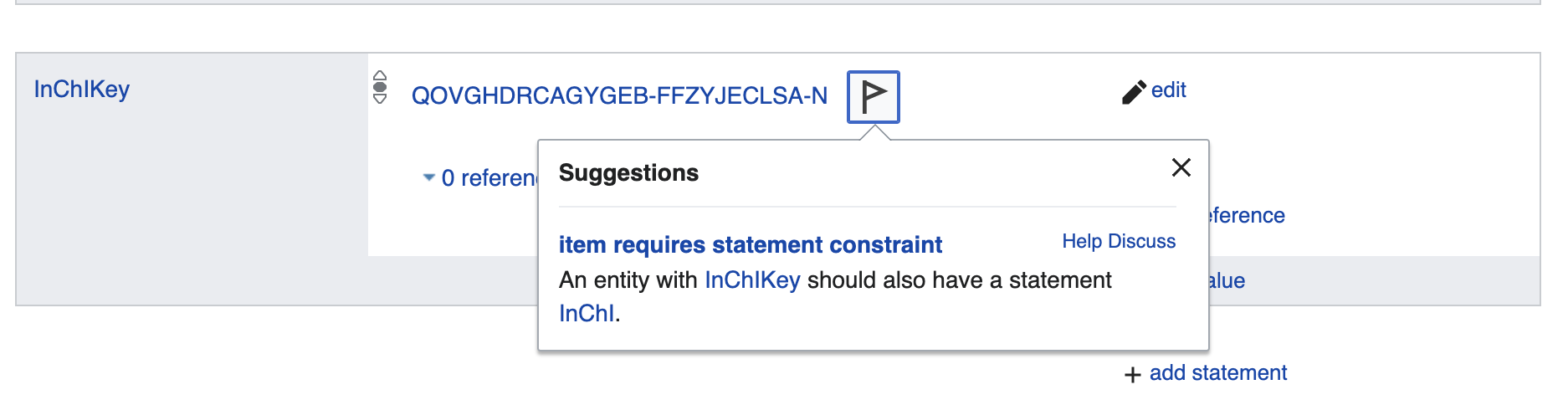


Here, Wikidata tells us that if we add an InChiKey, we will need to also add an InChI. Logical, but good to have a reminder!

Let’s go ahead and add the InChI string.

Likewise, the addition of an isomeric SMILES string will require us to add a Canonical SMILES.

Note that you might have to copy and paste the SMILES string from PubChem to a plain text editor and then back to Wikidata because of some formatting issues when copy pasting directly from PubChem.

C[C@@H]1C2[C@]([C@H]3[C@H]4[C@]1(C5=CC(=C(Cl)Cl)[C@@H]([C@]5([C@@H]([C@@]6([C@H]4O6)COC(=O)C)OC(=O)C)O)OC(=O)C7=CC=CC=C7)OC(O3)(O2)C8=CC=CC=C8)(C(=C)C)O

CC1C2C(C3C4C1(C5=CC(=C(Cl)Cl)C(C5(C(C6(C4O6)COC(=O)C)OC(=O)C)O)OC(=O)C7=CC=CC=C7)OC(O2)(O3)C8=CC=CC=C8)(C(=C)C)O

###### Add the biological source information

Now let’s add the found in taxon property ([P703](https://www.wikidata.org/wiki/Property:P703)).

Just click on Add a new statement and type in the first letters of the property you want to add:


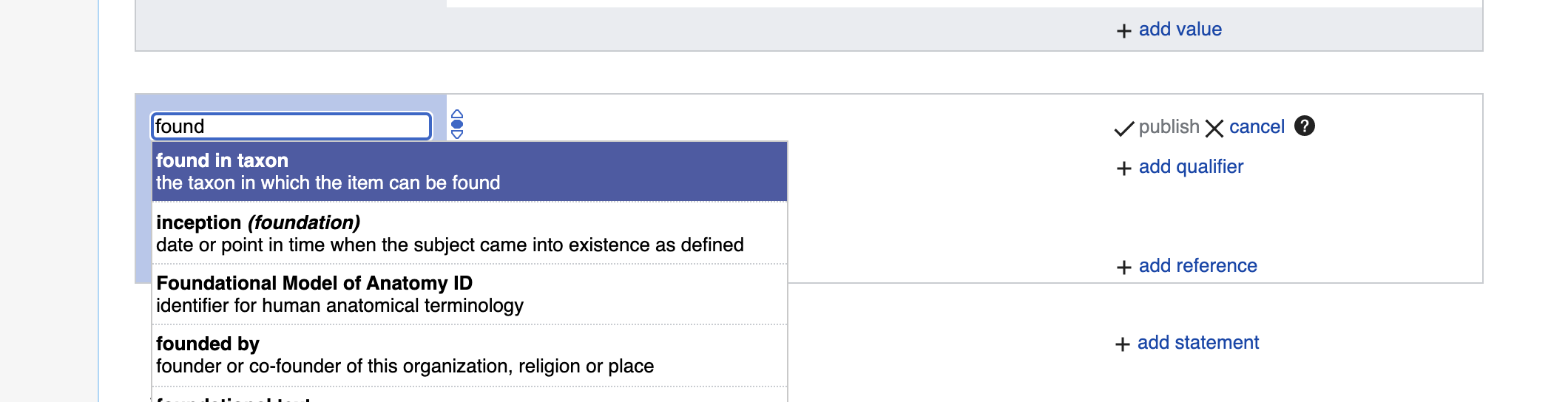


Again, type in the first letters of the taxon, and if the organism is present, it will autocomplete. Here is how this looks like for *Trigonostemon cherrieri*:


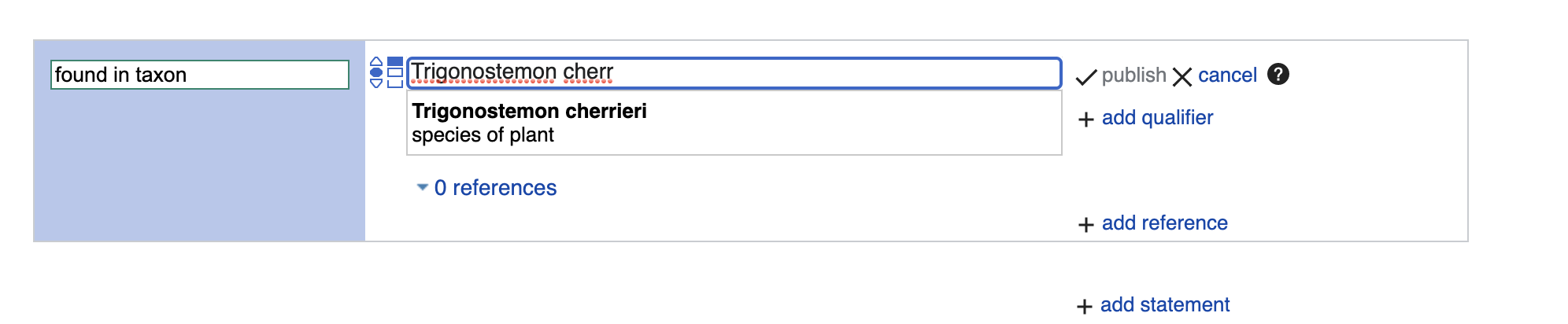


Click publish to save your changes and make them public.

If your target taxon is not yet present on Wikidata and you are sure you have a valid taxon name that is spelled correctly, then you can go to <https://www.wikidata.org/wiki/Special:NewItem>, as described in the [Add your data manually to Wikidata](#add_your_data_manually) section. For items about taxa, the instance of statement should have a value taxon (i.e. [Q16521](https://www.wikidata.org/wiki/Q16521)). As for chemical compounds, the user interface will then suggest to you further statements to add. For taxa, these include taxon name, parent taxon and taxon rank.

###### Add the reference documenting the structure-organism pair

Finally, since we report referenced structure-organisms pairs, we need to add the reference for this newly created compound found in taxon relationship. This happens on the item about the compound, just below the found in taxon statement. Click on the 0 references link and then on add reference:


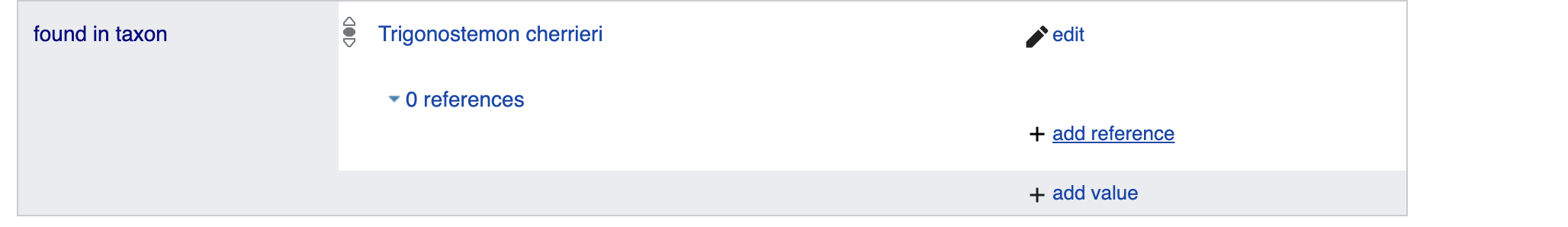


Here, we use the stated in property ([P248](https://www.wikidata.org/wiki/Property:P248)):


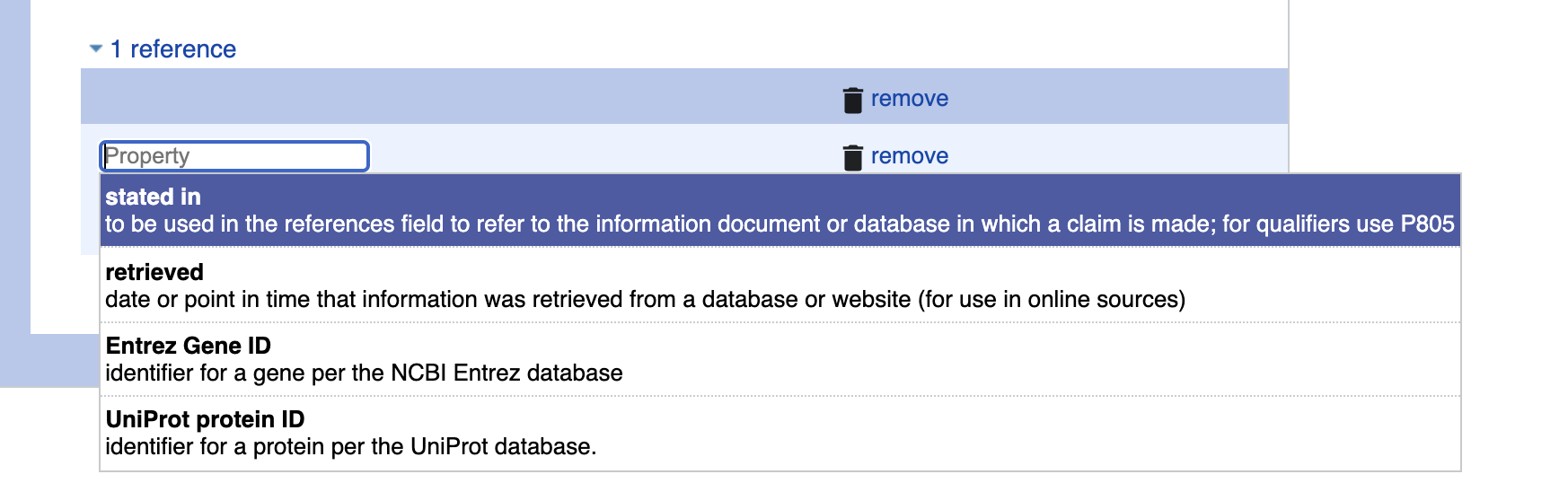


Now, type in the first letters or word of the scientific publication documenting the natural product occurrence, autocompletion happens again. Note that multiple publications might have the same title, and that there could be minor differences in punctuation or special characters between the information you and Wikidata have about the same reference. If you are not sure whether your target reference is already in Wikidata, you can use its DOI to check, as outlined in the [Check whether your target reference is already on Wikidata](#check_ref) section.


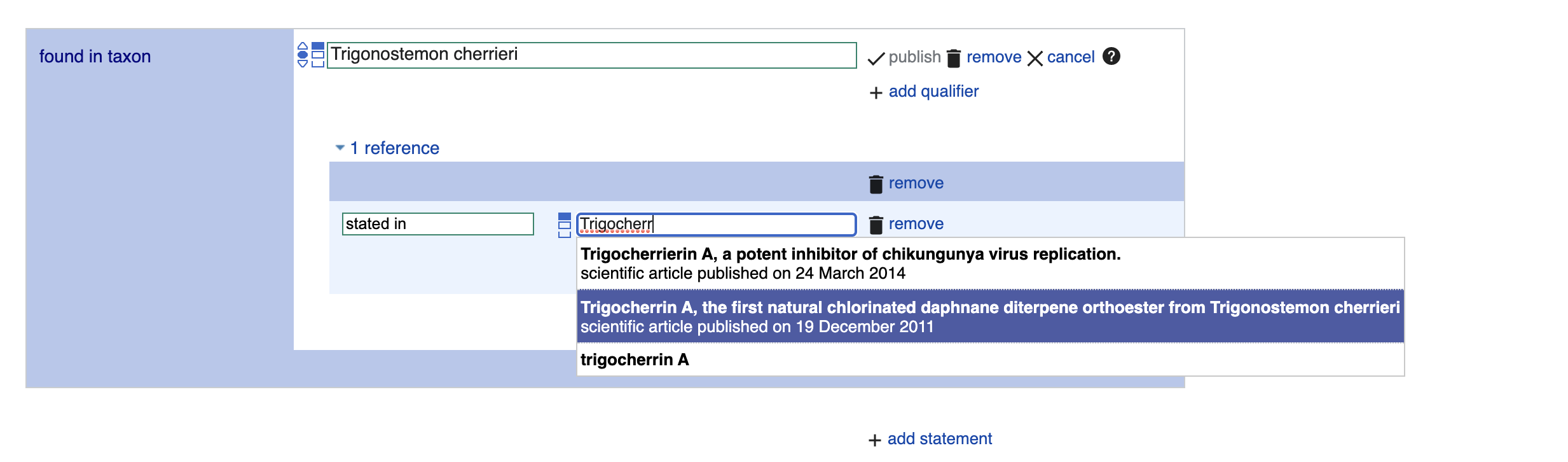


Click publish to save your changes and make them public.


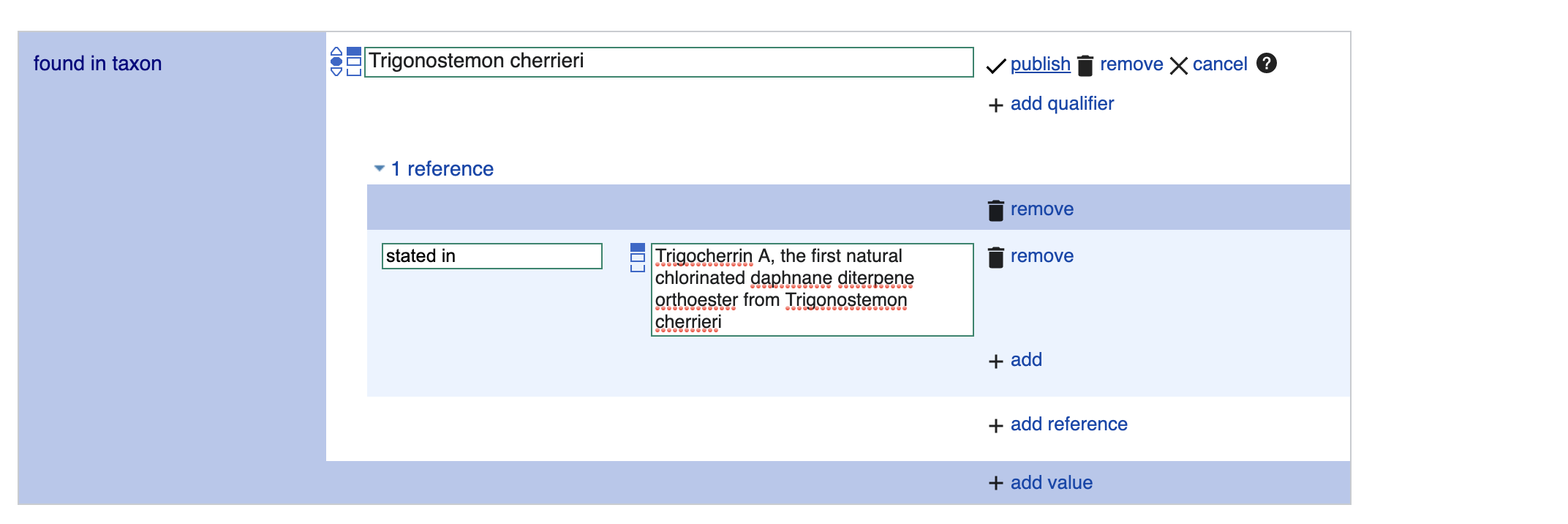


###### Check whether your target reference is already on Wikidata

If you are not sure whether your target reference is already in Wikidata, you can use its DOI to check. For our DOI 10.1021/ol2030907, the URL [https://scholia.toolforge.org/doi/***10.1021/ol2030907***](https://scholia.toolforge.org/doi/10.1021/ol2030907) will lead you to a [Scholia](https://www.wikidata.org/wiki/Wikidata:Scholia) page about this publication: <https://scholia.toolforge.org/work/Q83059010>. Scholia visualizes information from Wikidata, so if it has an entry for your target reference, then so does Wikidata, and both of them will use the same identifier (in this case [Q83059010](https://www.wikidata.org/wiki/Q83059010)). If you prefer to resolve your DOI to Wikidata directly, you can do so by using the uppercase-normalized DOI in the following URL pattern: [https://hub.toolforge.org/P356:**10.1021/OL2030907**](https://hub.toolforge.org/P356:10.1021/OL2030907), which will lead you to the respective Wikidata page, in this case [Q83059010](https://www.wikidata.org/wiki/Q83059010).

If you think that no Wikidata entry exists for your target reference, you can use the DOI in the URL pattern [https://sourcemd.toolforge.org/index_old.php?id=**10.1021/ol2030907**&doit=Check+source](https://sourcemd.toolforge.org/index_old.php?id=10.1021/ol2030907&doit=Check+source), which will trigger a check with both Crossref and Wikidata, and if no Wikidata entry can be found, the metadata from Crossref will be fetched and presented to you for creating the respective Wikidata item semi-automatically. Using such semi-automated workflows does require and account that is a minimum number of days old and has made a minimum number of edits on Wikidata.

If you are interested in the annotation of articles with topics via Wikidata and Scholia, here is a tutorial: <https://laurendupuis.github.io/Scholia_tutorial>.

##### Voilà !

You have added your first referenced structure-organism relationship to Wikidata and made a valuable contribution to the community. You can add further statements, e.g. molecular formula, or SPLASH code for linking to spectral data.

The Wikidata entry <https://www.wikidata.org/wiki/Q105674316> has been started using these instructions.

You can run a SPARQL query and check that everything went smoothly by modifying the InChiKey line in the following [SPARQL query](https://w.wiki/32zb):

SELECT ?item ?itemLabel ?taxonLabel ?artLabel WHERE {
 VALUES ?classes {
 wd:Q11173 # chemical compound
 wd:Q59199015 # group of stereoisomers
 wd:Q79529 # chemical substance
 wd:Q17339814 # group of chemical substances
 wd:Q47154513 # structural class of chemical compounds
 }
 ?item wdt:P31 ?classes. # instance of
 ?item wdt:P235 'QOVGHDRCAGYGEB-FFZYJECLSA-N' # InChiKey
 {
 ?item p:P1582 ?stmt. # natural product of taxon
 ?stmt ps:P1582 ?taxon. # natural product of taxon
 OPTIONAL {
 ?stmt prov:wasDerivedFrom ?ref.
 ?ref pr:P248 ?art. # stated in
 }
 }
 UNION
 {
 ?item p:P703 ?stmt. # found in taxon
 ?stmt ps:P703 ?taxon. # found in taxon
 OPTIONAL {
 ?stmt prov:wasDerivedFrom ?ref.
 ?ref pr:P248 ?art. # stated in
 }
 }
 SERVICE wikibase:label { bd:serviceParam wikibase:language "[AUTO_LANGUAGE],en". }
}
